# Can Repetitive Transcranial Magnetic Stimulation (rTMS) Promote Neurogenesis and Axonogenesis in Subacute Human Ischemic Stroke?

**DOI:** 10.3390/biomedicines12030670

**Published:** 2024-03-17

**Authors:** Manuela De Michele, Paola Piscopo, Matteo Costanzo, Svetlana Lorenzano, Alessio Crestini, Roberto Rivabene, Valeria Manzini, Luca Petraglia, Marta Iacobucci, Irene Berto, Oscar Gaetano Schiavo, Antonella Conte, Daniele Belvisi, Alfredo Berardelli, Danilo Toni

**Affiliations:** 1 Emergency Department, Stroke Unit, Policlinico Umberto I Hospital, Sapienza University, 00161 Rome, Italy; M.DeMichele@policlinicoumberto1.it (M.D.M.); luca.petraglia@uniroma1.it (L.P.); oscargaetano.schiavo@uniroma1.it (O.G.S.); 2 Department of Neuroscience, Italian National Institute of Health, 00161 Rome, Italy; paola.piscopo@iss.it (P.P.); matteo.costanzo@uniroma1.it (M.C.); alessio.crestini@iss.it (A.C.); roberto.rivabene@iss.it (R.R.); valemanzini44@gmail.com (V.M.); 3 Department of Human Neurosciences, Sapienza University, 00185 Rome, Italy; svetlana.lorenzano@uniroma1.it (S.L.); irene.berto@uniroma1.it (I.B.); daniele.belvisi@uniroma1.it (D.B.); alfredo.berardelli@uniroma1.it (A.B.); danilo.toni@uniroma1.it (D.T.); 4 Department of Biology and Biotechnology Charles Darwin, Sapienza University, 00185 Rome, Italy; 5 Neuroradiology Unit, Department of Human Neurosciences, Policlinico Umberto I Hospital, Sapienza University, 00161 Rome, Italy; marta.iacobucci@uniroma1.it; 6 IRCCS Neuromed, 86077 Pozzilli, Italy

**Keywords:** neurogenesis, stroke, humans, rTMS, miRNA, Netrin-1, semaphorin, MiR25, axonogenesis, ischemia

## Abstract

Background: Ischemic stroke may trigger neuroplastic changes via proliferation, migration towards the lesion, and differentiation of neuroprogenitor cells into mature neurons. Repetitive Transcranial Magnetic Stimulation (rTMS) may promote brain plasticity. This study aimed to assess rTMS’s effect on post-stroke endogenous neuroplasticity by dosing plasma miRs 17~92, Netrin-1, Sema3A, and BDNF. Methods: In this case-controlled study, we randomized 19 ischemic stroke patients within five days from symptoms onset (T0) to neuronavigated-rTMS or sham stimulation. Stimulation was applied on the stroke hemisphere daily between the 7th and 14th day from stroke onset. Blood samples were collected at T0, before the first rTMS section (T7), and at the end of the last rTMS session (T14). Five healthy controls were also enrolled in this study. Results: Of 19 patients, 10 received rTMS and 9 sham stimulation. Compared with the sham group, in the rTMS group, plasma levels of miRs17~92 and Ntn-1 significantly increased whereas Sema3A levels tended to decrease. In multivariate linear regression analyses, rTMS was independently related to Ntn-1 and miR-25 levels at T14. Conclusions: We found an association between rTMS and neurogenesis/axonogenesis biomarker enhancement. Our preliminary data suggest that rTMS may positively interfere with natural endogenous plasticity phenomena of the post-ischemic human brain.

## 1. Introduction

In most adult mammals, including humans, proliferating neural stem cells and neural progenitor cells (NPCs) are localized in the subventricular zone (SVZ) of the lateral ventricle wall and the subgranular zone (SGZ) of the hippocampal dentate gyrus [1,2,3,4,5]. Focal cerebral ischemia increases the proliferation of NPCs in both the SVZ and SGZ of rodents from the first four days to two weeks after stroke onset, which can persist for months [6,7,8,9,10]. Stroke-induced neurogenesis has also been demonstrated in human adult brain biopsies and autopsy specimens in the SVZ and ischemic boundary [11,12,13].

Transcranial magnetic stimulation (TMS) is a non-invasive technique that allows the stimulation of cortical motor areas through a coil placed over the scalp [14,15,16]. Single- and paired-pulse TMS protocols are now widely used to assess cortical excitability in patients with neurological disorders, including stroke, Parkinson’s disease, motor neuron diseases, and multiple sclerosis [15,16,17,18,19,20,21]. A few experimental studies using high-frequency rTMS have shown that HF-rTMS (5–20 Hz) can induce cell proliferation and enhance hippocampal neurogenesis in normal rat brains [22,23]. Notably, rTMS, delivered after experimental ischemic stroke in animal models, may enhance neurogenesis in the hippocampus through the Brain-Derived Neurotrophic Factor (BDNF) signaling pathway [24,25]. More recently, it has been demonstrated that HF-rTMS induces angiogenesis and neurogenesis, increases neuronal plasticity and perilesional tissue remodeling, and promotes axonal sprouting in a mouse stroke model [26]. To the best of our knowledge, no studies have so far evaluated whether rTMS can promote neurogenesis in patients with stroke. Current management guidelines for patients with stroke emphasize early intervention with thrombolytic agents, rehabilitation, and, in certain cases, surgical options. However, the limited window for therapeutic intervention and variable patient outcomes highlight the need for novel non-invasive therapeutic strategies for stroke survivors. In this context, HF-TMS might represents a potentially valuable therapeutic tool for stroke recovery.

MicroRNAs (miRs) are noncoding RNA molecules that modulate protein expression by binding to target mRNAs, and their involvement in rat ischemic brain damage has been reported [27]. The miR-17~92 family consists of three clusters (miR-17~92, miR-106b~25, and miR-106a~363) highly conserved among vertebrates [28,29]. MiR-25 seems to play a critical role in adult NPC proliferation [30], and the miR-106b~25 cluster appears to be the most expressed in the adult brain [30]. During brain and spinal cord development, guidance signals, including semaphorins and netrins, drive the formation of axons and vascular development. Semaphorin-3A (Sema3A) is a soluble repulsive signal leading to growth cone collapse [31], whereas Netrin-1 (Ntn-1) acts as a chemotropic/repulsive factor that mediates axon outgrowth, axon orientation, and neuronal migration during development [32]. Sema3A and Ntn-1 participate in post-stroke brain remodeling in rats [33,34].

The primary aim of the present study was to see whether HF-rTMS stimulation of cortical motor areas modulates peripheral biomarkers of neurogenesis and axogenesis in adult patients with subacute stroke. Specifically, to assess the effects of HF-rTMS on post-stroke neurogenesis, we measured plasmatic levels of miRs106b~25 cluster and BDNF. We also measured levels of Ntn-1 and Sema3A as peripheral markers for post-stroke axogenesis. For this study, the effects of a seven-day HF-rTMS intervention on peripheral blood markers in patients who had a stroke within five days prior were compared with the effect of HF-rTMS in a control group of patients with stroke who had sham HF-rTMS intervention. Blood biomarkers and TMS measures of cortical excitability, including the input/output curve, short-interval intracortical inhibition (SICI), and intracortical facilitation (ICF), were assessed at several points in time. Time points included a baseline evaluation (within 24 h post-stroke), a second evaluation before the start of the first rTMS session, and a final evaluation the day after completing the final rTMS session.

## 2. Materials and Methods

### 2.1. Patients

Nineteen non-consecutive patients (ten females; mean ± SD age 67.4 ± 14.7 years) and five HCs (three females; mean ± SD age 52.4 ± 23.9 years) were included in this case-controlled study from the 1 January to the 31 December 2020. Patients had an acute ischemic stroke of any vascular territory confirmed by MRI and were admitted to the stroke unit of our teaching hospital within five days of symptom onset. Exclusion criteria were as follows: lacunar stroke or large infarction (lesion volume larger than 1/3 of middle cerebral artery territory or half of the posterior cerebral artery territory); hemorrhagic transformation of the index infarct; patients with severe hemiparesis (National Institutes of Health Stroke Scale [NIHSS] motor item score ≥ 3) at 24 h from stroke onset, who were very likely to be transferred to a rehabilitation center and, hence, could not undergo 14-day rTMS; head injury; cancer; cerebral metastatic repetitions, central nervous system infections; unstable cardiac arrhythmia; epilepsy; seizures at onset of or after stroke; known neurodegenerative disease; and patients who were unable to give informed consent. Patients with any contraindication to MRI were also excluded. We collected demographic data for each patient, past medical history, vascular risk factors, and pre-stroke medications. Stroke severity and functional ability were assessed by using NIHSS and the modified Rankin Scale (mRS). Neurological examination and NIHSS scores were performed at admission to the emergency department and then again at 7 ± 2 and 14 ± 2 days. All clinical assessments, performed following standardized procedures, were carried out by a team of neurologists (MDM, LP, OGS, IB), from the Stroke Unit of the Emergency Department at Policlinico Umberto I Hospital, specialized in stroke treatment and care and trained in NIHSS scoring to ensure uniformity. A control group of 5 sex-matched healthy volunteers undergoing only one biomarker measurement and neuroimaging assessment was also included to compare their levels of miRs and the other molecular neurogenesis/axogenesis biomarkers with those of the patients measured at baseline (T0). Some authors (MDM, MC, LP, MI, IB, OGS) accessed information that could identify individual participants during and after data collection. All study participants gave written informed consent. Thus study was approved by the Ethical Committee of the Policlinico Umberto I Hospital (N 5473) and was conducted according to the Declaration of Helsinki.

### 2.2. Neuroimaging Techniques

All eligible patients underwent baseline 1.5 T multimodal Magnetic Resonance Imaging (MRI) (Magnetom Avanto, Siemens Healthineers, Muenchen, Germany) at the Emergency Department within 24 h from stroke onset. A 3.0 T MRI (Magnetom Verio, Siemens Healthineers, equipped with 12-channel head coil, Muenchen, Germany) was performed within 5 days of stroke onset, including the following imaging acquisitions: DWI with corresponding ADC mapping; three-dimensional (3D) T2-weighted sampling perfection with application-optimized contrasts by using flip angle evolution (SPACE) sequences and 3D-T1-weighted magnetization-prepared rapid gradient-echo (MPRAGE) sequences. The ischemic lesion was classified as cortical, subcortical, or cortical–subcortical, and the volume was calculated on the T2-FLAIR images obtained with the MRI performed within 24 h from stroke onset, through the A*B*C/2 formula, a semiquantitative method validated for ischemic stroke lesions [35]. The following parameters were used for the 3.0 T MRI: (I) TR/TE: 5000/70.6 ms, FOV: 230 × 230 mm, and diffusion gradients of b (0, 1000) s/mm^2^ for DWI; (II) TR/ TE: 5000/352 ms, FOV: 250 × 250 mm, matrix: 256 × 256, flip angle: 120°, imaging time: 3 min 42 s, 0.9 mm thick sections for 3D-T2 SPACE; (III) TR: 1740 ms, effective echo time (TE eff): 2.49 ms, inversion time: 900 ms, imaging time: 3 min 34 s, FOV: 240 × 240 mm, matrix: 256 × 256, and 0.9 mm thick sections for 3D-T1-MPRAGE.

### 2.3. TMS and Neurophysiological Measurements

**Figure 1:**
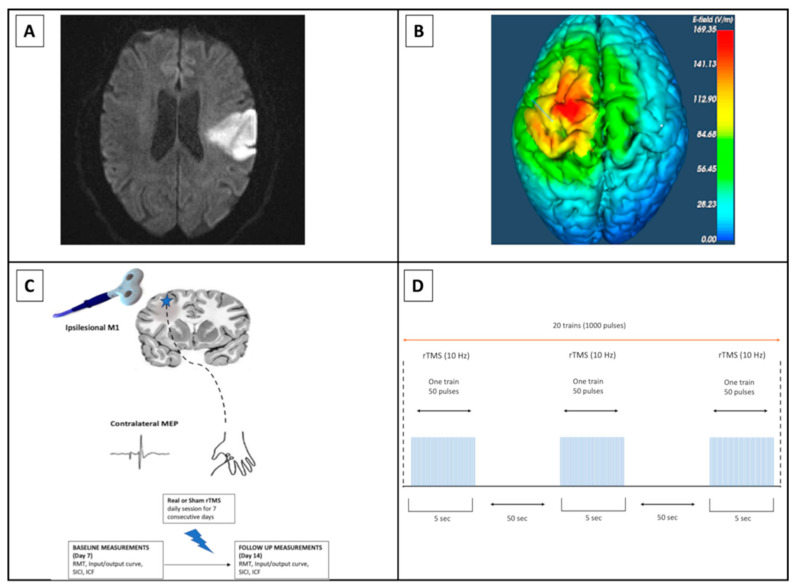
Transcranial Magnetic Stimulation protocol. (**A**) Diffusion-weighted imaging (DWI) of a representative patient with acute stroke in the left middle cerebral artery territory. The DWI sequence demonstrates the infarct lesion with an abnormally high signal (abnormal diffusion restriction). (**B**) Estimated Transcranial Magnetic Stimulation (TMS)-induced electric field of the left ipsilesional motor cortex (M1). (**C**) Schematic presentation of experimental design. A star symbol indicates the affected hemisphere. Single- and paired-pulse TMS was used to evaluate ipsilesional motor cortex (M1) excitability. Real or sham rTMS was delivered to the M1 area of the affected hemisphere. (**D**) Schematic presentation of rTMS excitatory protocol—10 Hz on M1 area of the affected hemisphere, using 100% of motor threshold: 20 trains, 50 pulses per train, 1000 pulses total.

Single and paired TMS paradigms were employed to assess cortical excitability in the two groups of patients (real and sham rTMS) participating in the study. Single- and paired-pulse TMS was delivered using a monophasic MAGSTIM 200 stimulator (MAGSTIM, Whitland, UK) connected to a figure-of-eight 70 mm diameter coil. The coil was held tangential to the scalp at an angle that induced a postero-anterior directed current perpendicular to the central sulcus. Magnetic stimuli were delivered over the affected primary motor cortex (M1) on the scalp position, eliciting the largest motor evoked potential (MEP) in the contralateral FDI muscle (i.e., hotspot) (Figure 1). When MEP was absent from the emisphere affected by the stroke, the motor “hot spot” was defined as symmetric to the non-stroke hemisphere. If MEPs appeared during recovery, the optimal site for stimulation of the stroke hemisphere was re-identified. RMT was defined as the minimum intensity required to elicit MEPs of ≥50 μV peak-to-peak amplitude in at least 5 out of 10 consecutive trials [36]. The input-output (I/O) curve was determined in each subject by measuring MEPs tested at intensity equal to 100%, 120%, and 140% RMT [37]. Ten MEPs were collected for each condition in randomized order. SICI and ICF were assessed using paired TMS with a subthreshold conditioning stimulus set at an intensity equal to 80% RMT, followed by a suprathreshold test stimulus at 120% RMT [17]. Interstimulus intervals (ISIs) between conditioning and test stimuli were set at 3 ms for SICI and 10 ms for ICF. Ten MEPs were collected for each ISI in randomized order. SICI and ICF were expressed as the percentage ratio between unconditioned and conditioned MEPs. I/O curve, SICI, and ICF were evaluated prior to the start of the first rTMS session (T7) and the day after completing the final rTMS session (T14). The rTMS intervention protocol consisted of stimulation for 5 s followed by rest for 50 s, which was repeated 20 times (1000 pulses per day) at 10 Hz (Figure 1). The stimulation intensity was set at 100% of the RMT of the unaffected hemisphere [16,38,39]. The subject’s head and the individual MR scan (T1WI 3D MPRAGE) were carefully co-registered in a common reference frame using an optic system for stereotaxic neuronavigation (Softaxic, EMS, Bologna, Italy). The neuronavigation system was used to monitor coil positioning during all TMS sessions (see Supplementary Materials for further details). Electromyographic (EMG) activity was recorded from the FDI muscle through pairs of Ag/AgCl surface electrodes placed in a belly–tendon montage. EMG signal was bandpass filtered 10–1000 Hz, amplified (×1000) (Digitimer D360; Digitimer, Welwyn Garden City, UK), digitized at 5 kHz (CED 1401; Cambridge Electronic Design, Cambridge, UK), and stored on a computer for offline analysis. MEP amplitudes were measured peak-to-peak and then averaged. Trials with involuntary EMG activity in the FDI muscle exceeding 100 uV in the 500 ms preceding the TMS pulse were rejected offline.

### 2.4. Biomarker Data

For the detection of miRs/BDNF/Ntn-1 and Sema3A levels, two venous blood samples (4 to 6 mL each) for each patient were collected in ethylenediaminetetraacetic acid (EDTA) tubes at T0 within 24 h of symptom onset. Subsequent blood samples were collected at the remaining time points (T7, at 7 ± 2 days after stroke onset, before starting the first rTMS session, and T14, at 14 ± 2 days at the end of the last rTMS session). For HCs, blood samples were collected only once before performing the 3 T MRI. All blood samples were immediately transferred to chilled, siliconized, disposable glass tubes. Hemolyzed blood samples were discarded. Plasma samples were obtained by blood centrifugation at 2500 rpm for 15 min, at 4 °C, aliquoted in 200 μL, and stored at −80 °C until assay.

Total plasma BDNF (MyBioSource, San Diego, CA, USA; catalog #: MBS824804), Ntn-1 (MyBioSource; catalog #: MBS700176), and Sema3A (MyBioSource; catalog #: MBS059778) concentrations were determined by specific enzyme-linked immunosorbent assay (ELISA), in accordance with the manufacturer’s protocol. Samples were analyzed in triplicate in blinded experiments, and their optical density was determined at 450 nm with a Tecan Sunrise microplate reader (Tecan, Männedorf, Switzerland). Results were obtained based on the relative standard curves.

MiRs extraction was performed by the miRNeasy Serum/Plasma Kit (Qiagen, Hilden, Germany), and reverse transcription into cDNA was performed using the miScript II RT Kit (Qiagen). A total of 1 μg of MS2 bacteriophage RNA was added to each sample to improve endogenous RNA recovery. Before the qRT-PCR, we monitored sample quality verifying possible cellular and hemolysis contaminations and possible carry-over of inhibitors into the RNA samples to successfully analyze miR expression, as previously reported. Total RNA was retro-transcribed using a Universal cDNA Synthesis kit (Qiagen). The cDNA template was then diluted 50× in nuclease-free water and mixed 1:1 with 2× PCR Master Mix (Qiagen). For miR-106b, miR-93, and miR-25 quantitative PCR assays, samples were analyzed in triplicate using the miRCURY PCR primers set, according to the miRCURY LNA SYBR green PCR Kit (Qiagen) manufacturer’s protocol. qRT-PCR reactions were performed by ABIPRISM 7500 Sequence detection system. Ct values were normalized using the Ct method with respect to the has-miR-16 endogenous control (dCT) [40,41,42].

### 2.5. Experimental Paradigm

**Figure 2:**
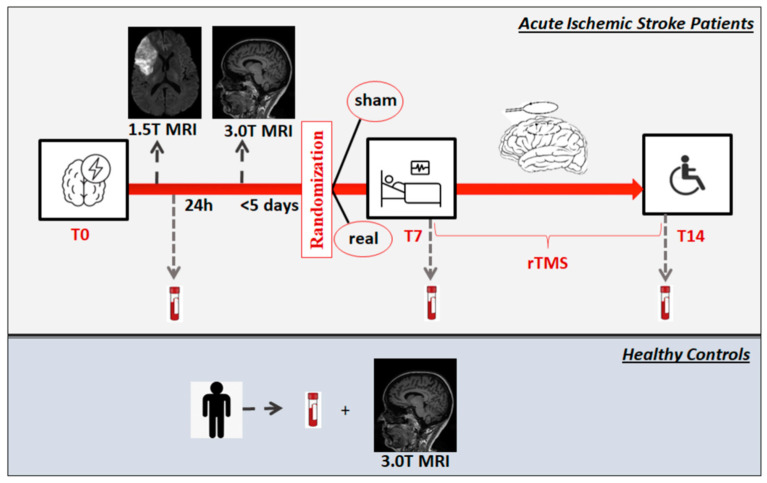
Schematic representation of the study experimental design. Patients with acute ischemic stroke were subjected to 1.5 T MRI within 24 h from onset. A 3.0 T MRI with 3D-T1- weighted magnetization-prepared rapid gradient-echo (MPRAGE) sequence was performed to allow the neuronavigation during all TMS sessions. Blood samples for biomarkers measurements were withdrawn within 24 h post-stroke (T0), before starting the first rTMS session (T7: 7 ± 2 days) and the day after completing the last rTMS session (T14: 14 ± 2 days). Patients were randomized to real or sham stimulation within T7 by closed envelopes. High-frequency rTMS was delivered daily for seven consecutive days from T7 to T14 per a prespecified protocol. Healthy controls underwent a 3.0 T MRI, and blood samples were collected for biomarkers analysis on the same day. MRI: Magnetic Resonance Imaging; T: Tesla or timepoint; rTMS repetitive Transcranial Magnetic Stimulation.

Patients were randomly assigned to two intervention groups with a 1:1 ratio using closed envelopes. The first group received real rTMS over the M1 area of the affected hemisphere, corresponding to the “hot spot” for the stimulation of the first dorsal interosseous (FDI) cortical area representation as defined during the resting motor threshold (RMT) determination; in contrast, the second group received sham stimulation of the same site (daily session for seven consecutive days from T7 to T14) (Figure 2). To minimize variability related to diurnal fluctuations in cortical excitability, TMS sessions for each participant were performed at the same time of day. The coordinator investigator of the study (MDM) generated the random allocation sequence. Some study investigators (MDM, LP, IB, OGS) enrolled participants and assigned them to interventions. Outcomes were the measurements of the following markers and their changes from before to after the intervention: (i) plasma miRs17~92 (miR-25, miR-106b) and BDNF, as potential peripheral markers of post-stroke human neurogenesis; (ii) plasma Netrin-1 and Sema3A as potential peripheral markers of post-stroke axogenesis.

### 2.6. Statistical Analysis

Statistical analysis was performed using SPSS statistical software (IBM Corp, SPSS Statistics for Windows, Version 25, Armonk, NY, USA). A formal sample size calculation was not performed due to the overall exploratory nature of this pilot study and because there was no specific literature data to use as a reference. Therefore, a sample of 20 patients (10 per intervention group) could be adequate to evaluate the primary and secondary objectives of the study. Descriptive data are presented as means ± SD (standard deviation) and/or median (interquartile range, IQR) and as percentages (with missing data excluded from the denominator) as appropriate. χ^2^ test, Fisher’s exact test, Student’s *t*-test, or the Mann–Whitney U test were used in the univariate analysis as appropriate based on the numbers of comparisons and distribution of the variables to compare demographics, baseline clinical, neuroimaging, rTMS, and biomarker data between patients undergoing real rTMS and those receiving sham rTMS and between patients and controls. We used one-way repeated measure ANOVA to compare the levels of neurogenesis-related molecular biomarkers across the different time points within the real and sham groups. A one-way ANOVA was also used to compare baseline neurophysiological parameters between patients assigned to the real stimulation and those assigned to the sham stimulation. Correlations were assessed by Spearman’s rank index coefficient (rho) to measure the statistical dependence between the rankings of two variables. Differences were considered significant at *p* < 0.05. An exploratory multivariate analysis was performed to evaluate whether rTMS independently predicts levels of neurogenesis/axogenesis-related molecular biomarkers at T14. Given the small number of patients included in the study, the risk of multivariate model overfitting should be considered, and the results should be interpreted cautiously. Variables with a univariate *p*-value < 0.05 were included in the multivariate models as well as the main clinical variables that can influence levels of blood biomarkers in stroke (age, sex, baseline NIHSS, lesion volume, acute treatments with IV thrombolysis [IVT], and/or mechanical thrombectomy [MT]). Three multivariate models were built: model 1 included the main prespecified demographics and clinical variables and baseline (T0) Ntn-1, miR25, or miR 106b levels; in model 2 and model 3, other biomarkers which at different timepoint significantly correlated with Ntn-1, miR25, or miR 106b levels at T14 were added to the variables included in model 1.

## 3. Results

### 3.1. Clinical Data

In the patient group, the most frequently reported vascular risk factor was arterial hypertension (*n* = 13, 68.4%); the median baseline (T0) NIHSS score was 3.0 (IQR 1.0–6.0), while it was 2.0 at both T7 (IQR 0–6.0) and T14 (IQR 0–5.0). Eight (42.1%) patients had a cortical–subcortical infarct, and seven (36.8%) patients had a cortical infarct; the baseline median (IQR) infarct volume was 5.40 (2.90–21.60). Overall, 8 (42.1%) patients received IV thrombolysis (IVT) and 6 (31.6%) patients underwent mechanical thrombectomy (MT) alone, while 3 (15.8%) patients received bridging therapy. Hemorrhagic transformation of the index infarct was detected in 6/18 (33.3%) patients (Supplementary Table S1). Of the 19 patients, 10 (50% females, mean ± SD age 70.9 ± 13.7 years) were randomized to real rTMS and 9 (55.6% females, mean ± SD age 63.4 ± 15.4 years) to sham stimulation. Demographics and baseline clinical characteristics by real, sham, and control groups are reported in Table 1. HCs had significantly lower median levels of BDNF and miR-106b than those of the real group at T0, higher median levels of Ntn-1 compared with the real and sham patients, and higher levels of miR93 than those of the sham group (*p* < 0.05 for all). No statistically significant differences were found between the real and sham groups in terms of age, sex, vascular risk factors, NIHSS total scores at the different time points (T0, T7, and T14), total leukocyte count at admission, infarct volume, infarct location, acute reperfusion treatments (IVT and/or MT), and hemorrhagic transformation of the index infarct (*p* > 0.05 for all variables). Arterial hypertension was numerically more frequent in the sham group compared with the real group (*n* = 8, 88.9% vs. *n* = 5, 50%; *p* = 0.141). No significant differences in age, sex, and vascular risk factors were observed between the control and real and sham groups, except for a significantly lower frequency of arterial hypertension in controls compared with sham patients (*n* = 1, 20% vs. *n* = 8, 88.9%; *p* = 0.023).

Plasma levels of neurogenesis-related molecular biomarkers at different time points by real, sham and control groups are reported in Table 2. There were no significant differences in the plasma biomarker levels between the real and sham groups at baseline (T0) and T7, except for higher median levels of Sema3A at T7 in the real rTMS patients (152.04 ng/mL vs. 50.25 ng/mL; *p* = 0.011). HCs had significantly lower median levels of BDNF and miR-106b than those of the real group at T0, higher median levels of Ntn-1 compared with the real and sham patients, and higher levels of miR93 than those of the sham group (*p* < 0.05 for all).

### 3.2. Neurophysiological Data

There were no adverse effects, and rTMS was well tolerated by all patients. Neurophysiological data are reported in Table 3. The values of RMT, I/O curve, SICI, and ICF before the start of the first session of rTMS were not significantly different between the two groups of patients (real vs. sham). No changes from T7 in the abovementioned neurophysiological parameters were observed at T14 (post-rTMS sessions) for both groups. Before the first session of rTMS, even at maximum stimulation output, the MEP from FDI was unobtainable in two patients, one in the real rTMS group and one in the sham group.

### 3.3. Plasma Biomarker Levels after rTMS Intervention

**Figure 3:**
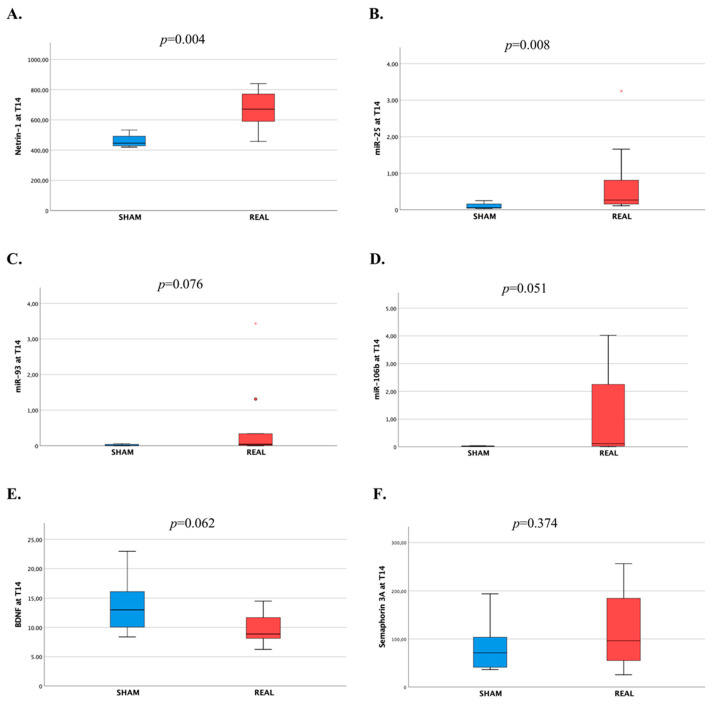
Levels of neurogenesis-related molecular biomarkers at T14 by real and sham groups. (**A**) Netrin-1. (**B**) miR-25. (**C**) miR-93. (**D**) miR-106b. (**E**) BDNF. (**F**). Semaphorin 3A. Boxes are drawn from the first quartile to the third quartile, horizontal lines are medians, vertical lines indicate maximum and minimum. Asterisks in (**B**,**C**) and the dot in (**C**) represent outliers. *p* values < 0.05 indicates statistically significant differences.

**Figure 4:**
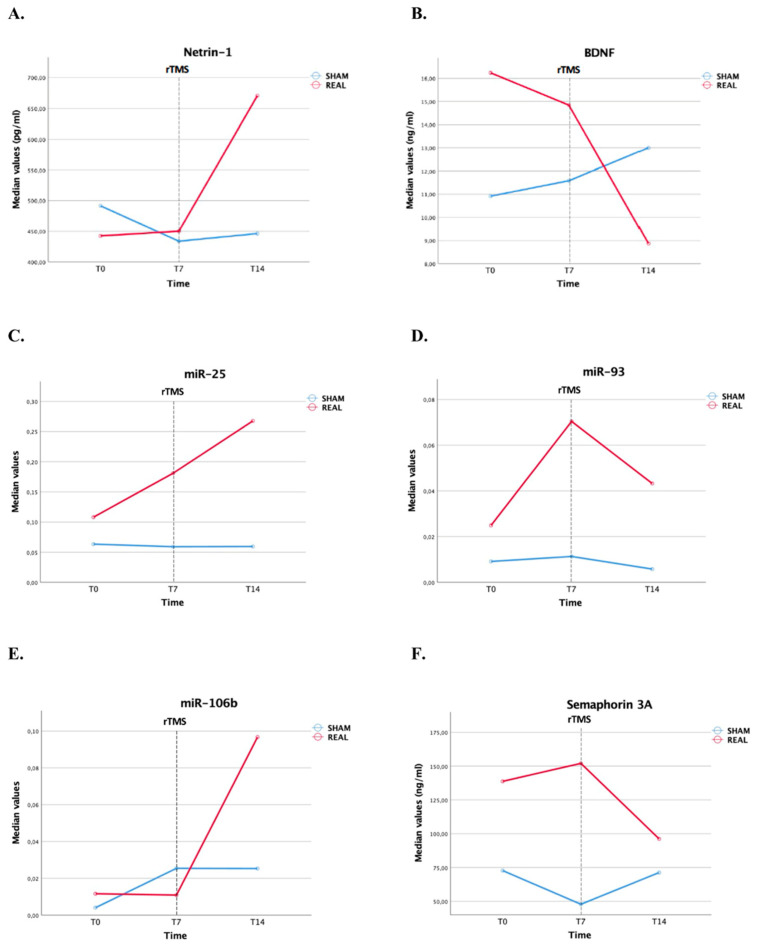
Temporal profile of median neurogenesis-related molecular biomarker levels across the different time points by real vs. sham groups (**A**) Netrin-1. (**B**) BDNF. (**C**) miR-25. (**D**) miR-93. (**E**) miR-106b. (**F**) Semaphorin 3A. Data on miRs are presented as 2-dCt where Ct is cycle threshold.

We observed statistically significant higher median levels of Ntn-1 in the real rTMS group at T14 compared with the sham group (671.00 pg/mL vs. 446.21 pg/mL; *p* = 0.004), as well as significantly higher levels of miR-25 (0.27 vs. 0.06; *p* = 0.008) and a borderline statistical significance for higher levels of miR-106b (0.11 vs. 0.03; *p* = 0.051) and miR-93 (0.04 vs. 0.01; *p* = 0.076) (Table 2 and Figure 3). Conversely, median levels of BDNF at T14 tended to be lower in the real rTMS group, although statistical significance was not reached (8.87 ng/mL vs. 13.0 ng/mL; *p* = 0.062) (Table 2 and Figure 3). Significant between-group differences in the changes of biomarker plasma levels at T14 (real vs. sham rTMS sessions) from T0 and T7 were consistently observed for Ntn-1, miR-25, and miR-106b (Supplementary Table S2 and Figure 4). Levels of Ntn-1 slightly increased in the sham group from T7 to T14, while they steeply increased in the real rTMS group at T14 from both T7 and T0 (*p* = 0.001 for both). This is also indicated by comparing the median plasma levels of Ntn-1 across the different time points within the groups (Supplementary Table S3). A similar steep increase in the real group was observed at T14 from both T7 and T0 for miR-25 (*p* = 0.001 for both) and miR-106b (ΔT14-T0, *p* = 0.027; ΔT14-T7, *p* = 0.004), compared with the sham group, for whom the levels of these miRs remained relatively stable at all time points. Conversely, levels of miR-93, after an initial increase from T0 to T7, decreased in the real group and remained relatively stable in the sham group (non-statistically significant difference). BDNF had a slight decrease in the real group from T0 to T7 followed by a steep reduction after rTMS from T7 to T14, while a progressive increase from T0 to T14 was observed in the sham group (ΔT7-T0, *p* = 0.022; ΔT14-T0, *p* = 0.026) (Supplementary Table S2 and Figure 4). This is also indicated by comparing the BDNF levels across the different time points within the groups (Supplementary Table S3). Sema3A levels, following an initial increase in the real group and a decrease in the sham group from T0 to T7, after rTMS reduced in the real group at T14 from T7, while they slightly increased in the sham group, but the between-group difference did not reach the statistical significance (Supplementary Table S2 and Figure 4).

Correlations of the neurogenesis-related molecular biomarkers among them and their associations with the demographics and clinical characteristics in all patients and in the real and sham groups are reported in Supplementary Tables S4–S6 and Figures S1–S5. In the exploratory multivariate linear regression analysis for Ntn-1 and miR-25 levels at T14 (Supplementary Materials), rTMS was the only independent predictor (Supplementary Table S7A,B). Conversely, rTMS does not seem to have an independent association with levels of miR-106b at T14 (Supplementary Table S7C).

## 4. Discussion

**Figure 5:**
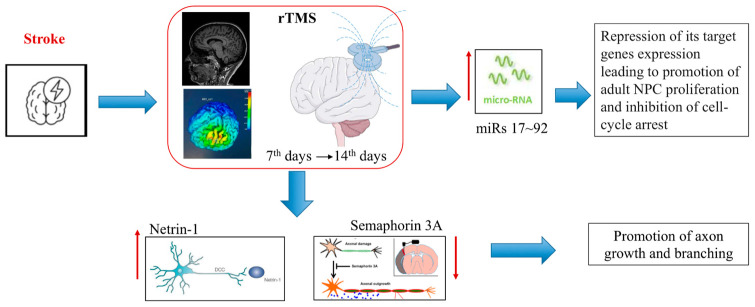
Schematic representation on how rTMS can modulate brain plasticity by promoting neurogenesis/axogenesis biomarkers.

This study investigated the effect of HF-rTMS on the plasmatic levels of neurogenesis/axonogenesis surrogate biomarkers in subacute ischemic stroke patients. We found that plasma miR-25 and Ntn-1 levels significantly increased in the rTMS-treated group, and a borderline statistical significance was found with higher levels of miR-106b and miR-93. HF-rTMS seems to independently predict higher levels of miR-25 and Ntn-1 after adjustment for confounding variables. These results may prove that rTMS could promote endogenous repair mechanisms (Figure 5).

Guo et al. demonstrated that adult neural stem cells increased in the rat ipsilateral SVZ 7 days after transient middle cerebral artery occlusion (MCAo) and that 10 Hz rTMS, delivered every 24 h for 7 days, promoted this proliferation [43]. Notably, the study showed that the level of miR-25 was significantly higher in the ipsilateral cortex after 10 Hz rTMS compared to the non-stimulated group. In the present study, we used a rTMS protocol that was similar to that used by Guo et al. in their experimental study [43] and, at the same time, followed the safety recommendations [16,44,45]. Our data measuring miR-25 in plasma from stroke patients align with Guo et al.’s observations in rat brains [43]. It is also important to note that serum miRs mirror their expression in the brain tissue [27]. Differently from Guo et al. [43], who showed a mildly increased expression of ischemic cortical miR-25 also in non-treated animals- suggesting that brain ischemia per se can stimulate miR-25 cellular expression- we did not find any statistically significant differences in the miR-25 levels at T0 and T7, between sham and real stimulated patients at baseline. It is possible that post-stroke non-stimulated miR-25 tissue levels could be too low at the different explored time points to become detectable in blood by the methodology used in our study. Another recent study confirmed the overexpression of miR-25 in the plasma of acute ischemic stroke patients and the ischemic brain tissue of rats subjected to MCAo and demonstrated a neuroprotective and neuroplasticity role for this miRNA [46].

We found a borderline statistical significance for higher levels of miR-106b and miR-93 at T14 in the real rTMS compared to sham rTMS patients. MiR-25, miR-106b and miR-93 all belong to the so-called miR-17~92 cluster (more specifically to its miR-106b~25 paralog), which largely contribute to vertebrate development and homeostasis, and recently it was found to be involved in tumorigenesis [47]. However, because of a different seed sequence inside the cluster, miR-25 seems to play a slightly different role than the two other miRNAs [47]. Brett et al. demonstrated that the miR-106b~25 cluster, mainly driven by miR-25, is likely to promote adult neural stem cell proliferation, whereas the entire cluster facilitates differentiation [30].

We have found a higher increase of Ntn-1 and a parallel downward trend of Sema3A in the real rTMS cohort. Furthermore, similarly to miR-25, in exploratory multivariate analyses, rTMS was an independent predictor of higher levels of Ntn-1. Ntn-1 is likely involved in axonal guidance (acting as an attractive guidance cue during cortical development) [48] with a neuroprotective role [32] promoting post-stroke neural function recovery by facilitation of synaptic formation and axonal regeneration [34], and it seems to induce rapid cortical axon branching [33].

We found a trend toward a steeper reduction of the BDNF levels after HF-rTMS compared to a slight increase from T0 to T14 in the sham group. BDNF belongs to a neurotrophin family of proteins with a wide range of potential roles in synaptic plasticity, neuronal growth, and neuronal survival [48]. A BDNF-mediated rTMS positive effect on functional recovery has been hypothesized. However, results from the literature on BDNF levels induced by rTMS are contradictory, and our data are in line with a recently published meta-analysis [49], which failed to demonstrate a serum increase of BDNF mediated by rTMS.

We found no changes in the neurophysiological parameters or significant clinical changes measured by the NIHSS at T14 between real and sham rTMS groups. However, this could be due to the mild stroke severity at baseline. Longer follow-up assessment may also be required for NPCs to differentiate into mature neurons and to integrate functionally into neuronal networks. Concordantly, stroke-induced newborn neurons have been electrophysiologically demonstrated to integrate into basal ganglia circuits 6–8 weeks after MCAo in rats [8]. On the contrary, the lack of differences in the neurophysiological variables between real and sham groups at T0 suggests that results from our study were not influenced by differences in cortical excitability at baseline between patients.

The main limitations of the present study are the relatively small number of patients investigated in our cohort and the relatively short follow-up in both real and sham rTMS groups. Furthermore, this is a single-center study; therefore, the results are not generalizable, and larger prospective randomized controlled studies are needed to confirm our results.

In conclusion, our findings provide new plausible evidence that HF-rTMS may modulate endogenous neurogenesis and axonal sprouting after ischemic stroke in humans. Our study opens up further prospects for research in this area. Future studies should aim to elucidate the precise mechanisms through which HF-rTMS influences neurogenesis and axonal sprouting, assess long-term outcomes, and determine the optimal parameters for treatment. Such advancements could significantly impact clinical practices by offering new, non-invasive treatment options for stroke survivors, ultimately contributing to improved recovery and quality of life.

## Figures and Tables

**Table 1:** Demographics and clinical characteristics by real, sham, and healthy control groups.

	Real *n* = 10	Sham *n* = 9	*p*	Healthy Controls *n* = 5	*p*
Age, mean (SD)	70.9 (13.7)	63.4 (15.4)	0.280 ^3^	52.4 (23.9)	vs. real: 0.125 ^3^ vs. sham: 0.310 ^3^
Sex, females; *n* (%)	5 (50)	5 (55.6)	1.0 ^1^	3 (60)	vs. real: 1.0 ^1^ vs. sham: 1.0 ^1^
Smoking; *n* (%)	2/9 (22.2)	4 (44.4)	0.620 ^1^	1 (20)	vs. real: 1.0 ^1^ vs. sham: 0.580 ^1^
Obesity; *n* (%)	3 (30)	2 (22.2)	1.0 ^1^	0	vs. real: 0.505 ^1^ vs. sham: 0.505 ^1^
Arterial hypertension; *n* (%)	5 (50)	8 (88.9)	0.141 ^1^	1 (20)	vs. real: 0.580 ^1^ vs. sham: **0.023** ^1^
Dyslipidemia; *n* (%)	3 (30)	3 (33.3)	1.0 ^1^	0	vs. real: 0.505 ^1^ vs. sham: 0.258 ^1^
Atrial fibrillation; *n* (%)	2 (20)	1 (11.1)	1.0 ^1^	0	vs. real: 0.524 ^1^ vs. sham: 1.0 ^1^
Diabetes mellitus; *n* (%)	0	2 (22.2)	0.211 ^1^	0	vs. real: - vs. sham: 0.505 ^1^
NIHSS, median (IQR);				-	-
mean (SD)					
- T0	4.50 (1.75–6.25);	3 (1–5);	0.282 ^2^		
	4.1 (2.3)	3 (2.6)			
- T7	3.50 (0.75–7.0);	2 (0–4);	0.297 ^2^		
	3.8 (3.1)	2.3 (3.0)			
- T14	3.50 (0.75–7.0);	2 (0–4);	0.166 ^2^		
	2.5 (2.9)	1.9 (2.1)			
Infarct volume, cm^3^, median (IQR)	6.50 (2.58–21.80)	5.40 (2.30–15.90)	0.624 ^2^	-	-
Infarct location; *n* (%)			0.744 ^1^	-	-
- cortical	3 (30)	4 (44.4)			
- subcortical	2 (20)	2 (22.2)			
- cortico-subcortical	5 (50)	3 (33.3)			
IVT; *n* (%)	5 (50)	6 (66.7)	0.650 ^1^	-	-
IVT alone; *n* (%)	5 (50)	3 (33.3)	0.650 ^1^	-	-
MT; *n* (%)	4 (40)	5 (55.6)	0.656 ^1^	-	-
MT alone; *n* (%)	4 (40)	2 (22.2)	0.628 ^1^	-	-
Bridging; *n* (%)	0	3 (33.3)	0.087 ^1^	-	-
HT of the index infarct; *n* (%)	2 (20)	4/8 (50)	0.321 ^1^	-	-
Total leukocytes at admission, mean (SD) (/mm^3^)	8806.0 (2456.9)	8836.7 (1427.9)	0.974 ^3^	-	-

Abbreviations: HT = hemorrhagic transformation; IQR = interquartile range; NIHSS = National Institutes of Health Stroke Scale; IVT = intravenous thrombolysis; MT = mechanical thrombectomy; SD = standard deviation. ^1^ Fisher’s exact test; ^2^ Mann-Whitney U test; ^3^ Student’s *t*-test. Bold indicates statistical significance.

**Table 2:** Neurogenesis-related molecular biomarker plasma levels at different time points by real, sham, and healthy control groups.

	Real *n* = 10	Sham *n* = 9	*p*	Healthy Control *n* = 5	*p*
Netrin-1, pg/mL, median					
(IQR)					
- T0	442.45	491.69	0.165	669.73	vs. real: **0.002**
	(422.31–495.88)	(472.95–495.46)		(631.92–774.71)	vs. sham: **0.003**
- T7	450.08	428.86	0.870	n.a	
	(407.34–481.07)	(415.15–477.36)			
- T14	671.00	446.21	**0.004**	n.a	
	(557.76–772.02)	(427.29–498.21)			
BDNF, ng/mL, median (IQR)					
- T0	16.24	11.49	0.050	9.80	vs. real: **0.014**
	(11.75–19.89)	(10.07–14.74)		(9.19–10.32)	vs. sham: 0.072
- T7	14.84	11.19	0.369		
	(7.35–16.49)	(10.60–14.35)			
- T14	8.87	13.0	0.062		
	(8.01–12.16)	(10.04–16.20)			
miR-25, median (IQR)					
- T0	0.11	0.06	0.288	0.08	vs. real: 0.624
	(0.04–0.60)	(0.04–0.12)		(0.07–0.11)	vs. sham: 0.386
- T7	0.18	0.05	0.221		
	(0.035–0.47)	(0.04–0.13)			
- T14	0.27	0.06	**0.008**		
	(0.15–1.02)	(0.04–0.19)			
miR-93, median (IQR)					
- T0	0.03	0.01	0.142	0.82	vs. real: 0.258
	(0.002–4.72)	(0.001–0.04)		(0.22–1.22)	vs. sham: **0.005**
- T7	0.07	0.05	0.165		
	(0.003–2.13)	(0.002–0.03)			
- T14	0.04	0.01	0.076		
	(0.009–0.58)	(0.002–0.05)			
miR-106b, median (IQR)					
- T0	0.01	0.002	0.102	0.0004	vs. real: **0.005**
	(0.004–0.23)	(0.0004–0.02)		(0.00005–0.0007)	vs. sham: 0.064
- T7	0.01	0.02	0.514		
	(0.005–0.27)	(0.002–0.04)			
- T14	0.11	0.03	0.051		
	(0.02–2.67)	(0.01–0.03)			
Semaphorin 3A, ng/mL, median					
(IQR)					
- T0	138.73	55.36	0.072	139.96	vs. real: 0.806
	(74.28–180.98)	(40.04–129.05)		(55.55–219.03)	vs. sham: 0.205
- T7	152.04	50.25	**0.011**		
	(68.30–184.69)	(43.12–112.68)			
- T14	96.17	71.24	0.374		
	(52.72–191.46)	(40.77–106.80)			

Abbreviations: BDNF = Brain Derived Growth Factor; HT = hemorrhagic transformation; IQR = interquartile range; SD = standard deviation. All comparisons were performed by using Mann-Whitney U test. Data on miRs are presented as 2-dCt where Ct is cycle threshold. Bold indicates statistical significance.

**Table 3:** Neurophysiological parameters before (at 7 days, T7) and after rTMS intervention (T14) in real and sham groups.

	Real rTMS Baseline *n* = 9	Sham rTMS Baseline *n* = 8	Post Real rTMS Sessions *n* = 9	Post Sham rTMS Sessions *n* = 8	*p* Value (Real rTMS vs. Sham rTMS Baseline)	*p* Value (Post Real rTMS Sessions vs. Post Sham rTMS Sessions)	*p*-Value (Real rTMS Baseline vs. Post Real rTMS sessions)	*p* Value (Sham rTMS Baseline vs. Post Sham rTMS Sessions)
MEPs amplitude 100% RMT (mV)	0.14 ± 0.09	0.08 ± 0.06	0.56 ± 1.02	0.5 ± 0.7	0.270	0.850	0.027	0.841
MEPs amplitude 120% RMT (mV)	1.1 ± 1.07	0.87 ± 0.82	1.78 ± 2.85	1.01 ± 1.08	0.792	0.794	0.397	0.999
MEPs amplitude 140% RMT (mV)	2.09 ± 1.72	1.94 ± 1.24	2.76 ± 2.66	1.48 ± 1.13	0.977	0.671	0.683	0.847
SICI value (%) *	33.3 ± 26.44	35 ± 33	57.7 ± 47.51	24.31 ± 21.36	0.776	0.222	0.245	0.69
ICF value (%) *	65 ± 83.8	87 ± 89	105.26 ± 132	31.93 ± 26.79	0.618	0.309	0.875	0.85

Abbreviations: MEPs = Motor evoked potentials; RMT = Resting motor threshold; SICI: Short-interval intracortical inhibition; ICF = Intracortical facilitation; rTMS: Repetitive transcranial magnetic stimulation. * SICI and ICF values were expressed as ratios, conditioned MEP/unconditioned MEP (in percentages).

## Data Availability

Data from the study are available upon reasonable request.

## Supplementary Materials

The following supporting information can be downloaded at: https://www.mdpi.com/article/10.3390/biomedicines12030670/s1, Table S1. Demographics and clinical characteristics of all study patients; Table S2. Changes of neurogenesis-related molecular biomarker plasma levels at T14 from baseline (T0) and T7 by real and sham groups; Table S3. Comparison of the levels of neurogenesis-related molecular biomarkers across the different timepoints within the real and sham groups; Table S4. Significant univariate correlations/associations for Netrin-1, miR25, and miR106b levels at T14 in all patients; Table S5. Statistically significant correlations among neurogenesis-related molecular biomarkers at different timepoints, in all patients and in the real and sham groups; Table S6. Statistically significant correlations among neurogenesis-related molecular biomarkers at different timepoints and demographics and clinical characteristics, in all patients and in the real and sham groups. Exploratory multivariate analysis: Results; Table S7. Multivariate linear regression analyses for Netrin-1, miR25, and miR106b levels at T14; Figure S1. Correlations between netrin-1 and mir25 at T0 (A) and T7 (B) in All patients (real + sham) and at T14 in real and sham (C) as separate subgroups; Figure S2. Correlations between mir25 and BDNF at T0 (A) and T7 (B) in All patients (real + sham) and at T14 in real and sham (C) as separate subgroups; Figure S3. Correlations among mir25, mir93, and mir106b at T0 (A) and T7 (B) in All patients (real + sham) and at T14 in real (C) and sham (D) subgroups; Figure S4. Correlations between netrin-1 and semaphorin3A as axonogenesis molecular biomarkers at T0 (A) and T7 (B) in All patients (real + sham) and at T14 in real (C) and sham (D) subgroups; Figure S5. Statistically significant correlations between age (A) and NIHSS at T7 (B) and molecular neurogenesis biomarkers (all with *p* < 0.05).
