# Supplementary material for: Can Repetitive Transcranial Magnetic Stimulation (rTMS) Promote Neurogenesis and Axonogenesis in Subacute Human Ischemic Stroke?
Source: Biomedicines. 2024 Mar 17;12(3):670. doi: 10.3390/biomedicines12030670 (PMC10968490; doi:10.3390/biomedicines12030670)

Supplementary material

Supplementary Table S1. Demographics and clinical characteristics of all study patients

	**All patients**<br>**N=19**
Age, mean (SD)	67.4 (14.7)
Sex, females (%)	10 (52.6)
Smoking (%)	6/18 (33.3)
Obesity (%)	5 (26.3)
Arterial hypertension (%)	13 (68.4)
Dyslipidemia (%)	6 (31.6)
Atrial fibrillation (5)	3 (15.8)
Diabetes mellitus (%)	2 (10.5)
NIHSS, median (IQR);<br>mean (SD)<br>- T0<br><br>- T7<br><br>- T14	 <br>3.0 (1.0-6.0);<br>3.6 (2.5)<br>2.0 (0-6.0);<br>3.1 (3.1)<br>2.0 (0-5.0);<br>2.8 (2.6)
Infarct volume, cm^3, median (IQR)	5.40 (2.90-21.60)
Infarct location (%)<br>- cortical<br>- subcortical<br>- cortico-subcortical	 7 (36.8)<br>4 (21.1)<br>8 (42.1)
IVT (%)	11 (57.9)
IVT alone (%)	8 (42.1)
MT (%)	9 (47.4)
MT alone (%)	6 (31.6)
Bridging (%)	3 (15.8)
HT of the index infarct (%)	6/18 (33.3)
Total leukocytes at admission, mean (SD) ($/mm^3$)	8820.5 (1981.1)

HT=hemorrhagic transformation; IQR=interquartile range; NIHSS=National Institutes of Health Stroke Scale; IVT=intravenous thrombolysis; MT=mechanical thrombectomy; SD=standard deviation.

Supplementary Table S2. Changes of neurogenesis-related molecular biomarker plasma levels at T14 from baseline (T0) and T7 by real and sham groups

	**Real**<br><br>**n=10**	**Sham**<br><br>**n=9**	**p**
Netrin-1, pg/ml, median (IQR)			
- ΔT7-T0	-17.91	-46.42	0.086
	(-25.96, -0.88)	(-62.22, 10.63)	
- ΔT14-T0	235.01	-41.61	**0.001**
	(119.06, 280.47)	(-58.69, 31.86)	
- ΔT14-T7	226.36	4.63	**0.001**
	(133.92, 304,40)	(-4.67, 23.81)	
BDNF, ng/ml, median (IQR)			
- ΔT7-T0	-3.32	-0.07	**0.022**
	(-3.90, -0.49)	(-1.14, 0.79)	
- ΔT14-T0	-6.07	0.57	**0.026**
	(-7.98, -3.82)	(-2.39, 5.08)	
- ΔT14-T7	-3.26	0.27	0.091
	(-7.31, 1.14)	(-1.48, 4.74)	
miR-25, ng/ml, median (IQR)			
- ΔT7-T0	0.007	0.003	0.935
	(-0.07, 0.07) 0.18	(-0.01, 0.02)	
- ΔT14-T0	(0.10, 0.49)	-0.002	**0.001**
	0.14	(-0.003, 0.024)	
- ΔT14-T7	(0.08, 0.54)	-0.001	**0.001**
		(-0.008, 0.020)	
miR-93, ng/ml, median (IQR)			
- ΔT7-T0	-0.001	0.001	0.514
	(-1.84, 0.04)	(-0.01, 0.01)	
- ΔT14-T0	-0.01	-0.001	0.722
	(-1.82, 0.05)	(-0.01, 0.002)	
- ΔT14-T7	-0.003	-0.003	0.657
	(-1.01, 0.04)	(-0.02, 0.02)	
miR-106b, ng/ml, median (IQR)			
- ΔT7-T0	-0.001	0.004	0.145
	(-0.01, 0.003)	(-0.001, 0.02)	

- ΔT14-T0	0.09<br>(0.01, 1.79)	0.01<br>(-0.003, 0.02)	**0.027**
- ΔT14-T7	0.08<br>(0.02, 1.73)	0.001<br>(-0.01, 0.01)	**0.004**
Semaphorin 3A, ng/ml, median (IQR)			
- ΔT7-T0	2.04<br>(-32.84, 27.85)	0.99<br>(-31.36, 14.33)	0.683
- ΔT14-T0	-15.34<br>(-108.87, 65.49)	-14.89<br>(-48.44, 0.64)	0.790
- ΔT14-T7	-17.01<br>(-111.97, 64.01)	-6.95<br>(-28.35, 19.08)	0.477

IQR=interquartile range

Supplementary Table S3. Comparison of the levels of neurogenesis-related molecular biomarkers across the different timepoints within the real and sham groups

	T0	T7	T14	p
**Netrin-1 (pg/ml)**				
- Real	442.45<br>(422.31-495.88)	450.08<br>(407.34-481.07)	671.00<br>(557.76-772.02)	**0.001***<br>T7 vs T0: 1.0; **T14 vs T0: 0.001**; **T14 vs T7: 0.001**
- Sham	491.69<br>(472.95-495.46)	428.86<br>(415.15-477.36)	446.21<br>(427.29-498.21)	0.066*<br>T7 vs T0: 0.169; T14 vs T0: 1.0; T14 vs T7: 0.792
**BDNF (ng/ml)**				
- Real	16.24<br>(11.75-19.89)	14.84<br>(7.35-16.49)	8.87<br>(8.01-12.16)	0.065*<br>T7 vs T0: 0.137; T14 vs T0: 0.130; T14 vs T7: 0.820
- Sham	11.49<br>(10.07-14.74)	11.19<br>(10.60-14.35)	13.0<br>(10.04-16.20)	0.777<br>T7 vs T0: 1.0; T14 vs T0: 1.0; T14 vs T7: 1.0
**mir25**				
- Real	0.11<br>(0.04-0.60)	0.18<br>0.035-0.47)	0.27<br>(0.15-1.02)	0.215*<br>T7 vs T0: 1.0; T14 vs T0: 0.216; T14 vs T7: 0.232
- Sham	0.06<br>(0.04-0.12)	0.05<br>(0.04-0.13)	0.06<br>(0.04-0.19)	0.618*<br>T7 vs T0: 1.0; T14 vs T0: 0.926; T14 vs T7: 1.0

**mir93**				
- Real	0.03 (0.002-4.72)	0.07 (0.003-2.13)	0.04 (0.009-0.58)	0.365* T7 vs T0: 0.728; T14 vs T0: 0.621; T14 vs T7: 0.431
- Sham	0.01 (0.001-0.04)	0.05 (0.002-0.03)	0.01 (0.002-0.05)	0.870* T7 vs T0: 1.0; T14 vs T0: 1.0; T14 vs T7: 1.0
**mir106b**				
- Real	0.01 (0.004-0.23)	0.01 (0.005-0.27)	0.11 (0.02-2.67)	0.196* T7 vs T0: 1.0; T14 vs T0: 0.279; T14 vs T7: 0.259
- Sham	0.002 (0.0004-0.02)	0.02 (0.002-0.04)	0.03 (0.01-0.03)	0.115* T7 vs T0: 0.268; T14 vs T0: 0.186; T14 vs T7: 1.0
**Semaphorin 3A**				
- Real	138.73 (74.28-180.98)	152.04 (68.30-184.69)	96.17 (52.72-191.46)	0.800* T7 vs T0: 1.0; T14 vs T0: 1.0; T14 vs T7: 1.0
- Sham	55.36 (40.04-129.05)	50.25 (43.12-112.68)	71.24 (40.77-106.80)	0.485* T7 vs T0: 0.746; T14 vs T0: 1.0; T14 vs T7: 1.0

Values are median (IQR); T0=baseline; T7=7 days from stroke onset; T14=14 days from stroke.
BDNF=Brain-derived growth factor
***Results from the one-way repeated measured ANOVA:**
REAL: Netrin-1: Wilks' Lambda 0.19, $F(2, 8) = 16.80$, p=0.001, $\eta^2 = 0.80$; **BDNF:** Wilks' Lambda 0.50, $F(2, 8) = 3.93$, p=0.065, $\eta^2 = 0.50$; **mir25:** Wilks' Lambda 0.78, $F(2, 8) = 1.87$, p=0.215, $\eta^2 = 0.32$; **mir93:** Wilks' Lambda 0.68, $F(2, 8) = 1.15$, p=0.365, $\eta^2 = 0.22$; **mir106b:** Wilks' Lambda 0.63, $F(2, 7) = 2.08$, p=0.196, $\eta^2 = 0.37$; **SemaphorinA:** Wilks' Lambda 0.95, $F(2, 8) = 0.23$, p=0.196, $\eta^2 = 0.05$.
SHAM: Netrin-1: Wilks' Lambda 0.40, $F(2, 6) = 4.42$, p=0.066, $\eta^2 = 0.60$; **BDNF:** Wilks' Lambda 0.92, $F(2, 6) = 0.26$, p=0.777, $\eta^2 = 0.08$; **mir25:** Wilks' Lambda 0.85, $F(2,6) = 0.52$, p=0.618, $\eta^2 = 0.15$; **mir93:** Wilks' Lambda 0.96, $F(2, 6) = 0.14$, p=0.870, $\eta^2 = 0.05$; **mir106b:** Wilks' Lambda 0.49, $F(2, 6) = 3.16$, p=0.115, $\eta^2 = 0.51$; **SemaphorinA:** Wilks' Lambda 0.79, $F(2, 8) = 0.82$, p=0.485, $\eta^2 = 0.21$.

Supplementary Table S4. Significant univariate correlations/associations for Netrin-1, miR25, and miR106b levels at T14 in all patients

	Real vs. sham rTMS	Netrin-1 T14	BDNF T0	mir25 T0	mir25 T7	mir25 T14	mir93 T0	mir93 T7	mir93 T14	mir106b T0	mir106b T7	mir106b T14	Semaphorin 3A T0	Semaphorin 3A T7	Hypertension yes vs. no	NIHSS T7	NIHSS T14
**Netrin-1 T14**	671.0 vs.<br>446.21<br>0.004	-	0.469<br>0.050	-	0.530<br>0.024	0.640<br>0.004	-	-	0.629<br>0.005	-	-	0.505<br>0.033	0.506<br>0.032	0.520<br>0.027	459.51 vs<br>771.12<br>0.005	-	-
**mir25 T14**	0.27 vs.<br>0.06<br>0.008	0.640<br>0.004	0.513<br>0.030	0.781<br><0.001	0.777<br><0.001	-	-	-	-	0.534<br>0.027	-	0.664<br>0.003	0.503<br>0.034	0.736<br>0.001	-	0.595<br>0.009	0.583<br>0.011
**miR106b T14**	0.11 vs.<br>0.03<br>0.051	0.505<br>p=0.033	-	-	-	0.664<br>p=0.003	0.575<br>p=0.013	0.697<br>p=0.001	-	0.711<br>p=0.001	0.635<br>p=0.005	-	0.474<br>p=0.047	-	-	-	-

Numbers are i) for continuous variables, Spearman's correlation coefficient (rho) and p values; ii) for dichotomous /categorical variables, median values and p values

Supplementary Table S5. Statistically significant correlations among neurogenesis-related molecular biomarkers at different timepoints, in all patients and in the real and sham groups

	Netrin-1 T0	Netrin-1 T7	Netrin-1 T14	BDNF T0	BDNF T7	BDNF T14	mir25 T0	mir25 T7	mir25 T14	mir93 T0	mir93 T7	mir93 T14	mir106b T0	mir106b T7	mir106b T14	Semaphorin3A T0	Semaphorin3A T7	Semaphorin3A T14
**Netrin-1 T0**																		
**- All**	-	0.609<br>0.006	-	-	-	0.560<br>0.016	-	-	-	-	-	-	-	-	-	-	-	-
**- Real**	-	0.661<br>0.038	-	-	-	-	-	-	-	-	-	-	-	-	-	-	-	-
**- Sham**	-	0.750<br>0.020	-	-	-	-	-	-	-	-	-	-	-	-	-	-	-	-
**Netrin-1 T7**																		
**- All**	0.609<br>0.006	-	-	-	-	-	-	-	-	-	-	-	-	-	-	-	-	-
**- Real**		-	0.709<br>0.022	-	-	-	-	-	-	-	-	-	-	-	-	-	-	-
**- Sham**	0.750<br>0.020	-	-	-	-	-	-	-	-	-	-	-	-	-	-	-	-	-
**Netrin-1 T14**																		
**- All**	-	-	-	0.469<br>0.050	-	-	-	0.530<br>0.024	0.640<br>0.004	-	-	0.629<br>0.005	-	-	0.505<br>0.033	0.506<br>0.032	0.520<br>0.027	-
**- Real**	-	0.709<br>0.022	-	-	-	0.806<br>0.005	0.745<br>0.013	0.733<br>0.016	-	-	-	-	-	-	-	0.782<br>0.008	-	-
**- Sham**	-	0.762<br>0.028	-	-	-	-	-	-	-	-	-	-	-	-	-	-	-	-
**BDNF T0**																		
**- All**	-	-	0.469<br>0.050	-	0.768<br><0.001	-	0.477<br>0.039	0.523<br>0.022	0.513<br>0.030	-	-	0.531<br>0.023	-	-	-	-	0.493<br>0.032	-
**- Real**	-	-	-	-	0.685<br>0.029	-	-	-	-	-	-	0.758<br>0.011	-	-	-	-	-	-
**- Sham**	-	-	-	-	-	-	-	-	-	-	-	-	-	-	-	-	-	-
**BDNF T7**																		
**- All**	-	-	-	0.768<br><0.001	-	-	0.581<br>0.009	0.651<br>0.003	-	0.502<br>0.029	-	0.519<br>0.027	-	-	-	-	0.474<br>0.040	-
**- Real**	-	-	0.762<br>0.028	0.685<br>0.029	-	-	-	-	-	-	-	-	-	-	-	-	-	-
**- Sham**	-	-	-	-	-	-	-	-	-	-	-	-	-	-	-	-	-	-
**BDNF T14**																		
**- All**	0.560<br>0.016	-	-	-	-	-	-	-	-	-	-	-	-	0.550<br>0.018	-	-	-	-
**- Real**	-	-	0.806<br>0.005	-	0.661<br>0.038	-	-	-	-	-	-	-	-	0.929<br>0.001	-	0.709<br>0.022	-	-

**- Sham**	-	-	-	-	-	-	-	-	-	-	-	-	-	-	-	-	-	-
**mir25 T0**<br>**- All**	-	-	-	0.477<br>0.039	0.581<br>0.009	-	-	0.961<br><0.001	0.781<br><0.001	-	0.512<br>0.025	0.515<br>0.029	0.478<br>0.045	0.539<br>0.017	-	0.474<br>0.040	0.496<br>0.031	-
**- Real**	-	-	0.745<br>0.013	-	-	-	-	0.952<br><0.001	0.891<br><0.001	-	0.758<br>0.011	0.842<br>0.002	-	-	-	0.709<br>0.022	0.721<br>0.019	-
**- Sham**	-	-	-	-	-	-	-	0.833<br>0.002	0.976<br><0.001	-	-	-	-	-	-	-	-	-
**mir25 T7**<br>**- All**	-	-	0.530<br>0.024	0.523<br>0.022	0.651<br>0.003	-	0.961<br><0.001	-	0.777<br><0.001	-	0.493<br>0.002	0.544<br>0.020	-	0.514<br>0.024	-	0.589<br>0.008	0.581<br>0.009	-
**- Real**	-	-	0.733<br>0.016	-	-	0.661<br>0.038	0.952<br><0.001	-	0.879<br>0.001	-	0.709<br>0.022	0.818<br>0.004	-	0.636<br>0.048	0.758<br>0.011	0.794<br>0.006	-	-
**- Sham**	-	-	-	-	-	-	0.883<br>0.002	-	0.905<br>0.002	-	-	-	-	-	-	-	-	-
**mir25 T14**<br>**- All**	-	-	0.640<br>0.004	0.513<br>0.030	-	-	0.781<br><0.001	0.777<br><0.001	-	-	-	-	0.534<br>0.027	-	0.664<br>0.003	0.503<br>0.034	0.736<br>0.001	-
**- Real**	-	-	-	-	-	-	0.891<br>0.001	0.879<br>0.001	-	0.782<br>0.008	0.891<br>0.001	0.697<br>0.025	0.750<br>0.020	0.830<br>0.003	0.721<br>0.019	0.721<br>0.019	0.855<br>0.002	-
**- Sham**	-	-	-	-	-	-	0.976<br><0.001	0.905<br>0.002	-	-	-	-	-	-	-	-	-	-
**mir93 T0**<br>**- All**	-	-	-	-	0.502<br>0.029	-	-	-	-	-	0.854<br><0.001	0.738<br><0.001	0.773<br><0.001	0.868<br><0.001	0.575<br>0.013	-	-	-
**- Real**	-	-	-	-	-	-	-	-	0.782<br>0.008	-	0.915<br><0.001	-	0.917<br>0.001	0.891<br>0.001	0.842<br>0.002	0.648<br>0.043	0.648<br>0.043	-
**- Sham**	-	-	-	-	-	-	-	-	-	-	0.800<br>0.010	0.810<br>0.015	-	0.683<br>0.042	-	-	-	-
**mir93 T7**<br>**- All**	-	-	-	-	-	-	0.512<br>0.025	0.493<br>0.032	-	0.854<br><0.001	-	0.657<br>0.003	0.796<br><0.001	0.753<br><0.001	0.697<br>0.001	-	-	-
**- Real**	-	-	-	-	-	-	0.758<br>0.011	0.709<br>0.022	0.891<br>0.001	0.951<br><0.001	-	0.745<br>0.015	0.867<br>0.002	0.927<br><0.001	0.830<br><0.003	0.709<br>0.022	0.745<br>0.013	-
**- Sham**	-	-	-	-	-	-	-	-	-	0.800<br>0.010	-	-	0.700<br>0.036	-	-	-	-	-
**mir93 T14**<br>**- All**	-	-	0.629<br>0.005	0.531<br>0.023	0.519<br>0.027	-	0.515<br>0.029	0.544<br>0.020	-	0.738<br><0.001	0.657<br>0.003	-	-	-	-	-	-	-
**- Real**	-	-	-	0.758<br>0.011	-	-	0.842<br>0.002	0.818<br>0.004	0.697<br>0.025	-	0.745<br>0.013	-	-	-	-	0.782<br>0.008	-	-
**- Sham**	-	-	-	-	-	-	-	-	-	0.810<br>0.015	-	-	-	-	-	-	-0.714<br>0.047	-
**mir106b T0**																		

**- All**	-	-	-	-	-	-	0.478<br>0.045	-	0.534<br>0.027	0.773<br><0.001	0.796<br><0.001	-	-	0.730<br>0.001	0.711<br>0.001	-	-	-
**- Real**	-	-	-	-	-	-	-	-	0.750<br>0.020	0.917<br>0.001	0.867<br>0.002	-	-	0.933<br><0.001	0.833<br>0.005	-	0.700<br>0.036	-
**- Sham**	-	-	-	-	-	-	-	-	-	-	0.700<br>0.036	-	-	-	-	-	-	-
**mir106b T7**<br>**- All**	-	-	-	-	-	0.550<br>0.018	0.539<br>0.017	0.514<br>0.024	-	0.868<br><0.001	0.755<br><0.001	0.610<br>0.007	0.730<br>0.001	-	0.635<br>0.005	-	-	-
**- Real**	-	-	-	-	-	-	0.709<br>0.022	0.636<br>0.048	0.830<br>0.003	0.891<br>0.001	0.927<br><0.001	-	-	0.933<br><0.001	0.915<br><0.001	-	0.661<br>0.038	-
**- Sham**	-	-	-	-	-	0.929<br>0.001	-	-	-	0.683<br>0.042	-	-	-	-	-	-	-	-
**mir106b T14**<br>**- All**	-	-	0.505<br>0.033	-	-	-	-	-	0.664<br>0.003	0.575<br>0.013	0.697<br>0.001	-	0.711<br>0.001	0.635<br>0.005	-	0.474<br>0.047	-	-
**- Real**	-	-	-	-	-	-	-	-	0.721<br>0.019	0.842<br>0.002	0.830<br>0.003	-	0.833<br>0.005	0.915<br><0.001	-	-	-	-
**- Sham**	-	-	-	-	-	-	-	-	-	-	-	-	-	-	-	-	-	-
**Semaforion3A T0**<br>**- All**	-	-	0.506<br>0.032	-	-	-	0.474<br>0.040	0.589<br>0.008	0.503<br>0.034	-	-	-	-	-	0.474<br>0.047	-	0.714<br>0.001	-
**- Real**	-	-	0.782<br>0.008	-	-	0.709<br>0.022	0.709<br>0.022	0.758<br>0.011	0.721<br>0.019	0.648<br>0.043	0.709<br>0.022	0.782<br>0.008	-	-	-	-	-	-
**- Sham**	-	-	-	-	-	-	-	-	-	-	-	-	-	-	-	-	0.817<br>0.007	-
**Semaphorin3A T7**<br>**- All**	-	-	0.520<br>0.027	0.493<br>0.032	0.474<br>0.040	-	0.496<br>0.031	0.581<br>0.009	0.736<br>0.001	-	-	-	-	-	-	0.714<br>0.001	-	-
**- Real**	-	-	-	-	-	-	0.721<br>0.019	0.794<br>0.006	0.855<br>0.002	0.648<br>0.043	0.745<br>0.013	-	0.700<br>0.036	0.661<br>0.038	-	-	-	-
**- Sham**	-	-	-	-	-	-	-	-	-	-	-	0.714<br>0.047	-	-	-	0.817<br>0.007	-	-
**Semaphorin3A T14**<br>**- All**	-	-	-	-	-	-	-	-	-	-	-	-	-	-	-	-	-	-
**- Real**	-	-	-	-	-	-	-	-	-	-	-	-	-	-	-	-	-	-
**- Sham**	-	-	-	-	-	-	-	-	-	-	-	-	-	-	-	-	-	-

Numbers are Spearman’s correlation coefficient (rho) and p values

Supplementary Table S6. Statistically significant correlations among neurogenesis-related molecular biomarkers at different timepoints and demographics and clinical characteristics, in all patients and in the real and sham groups.

	Age	Sex	Obesity	Smoking	Hypertension	NIHSS	Leukocytes at admission	IVT	Thrombectomy	Thrombectomy alone	Bridging	Lesion location
**Netrin-1 T0**<br>**- All**<br>**- Real**<br>**- Sham**	-	-	-	-	-	-	-	-	-	-	-	-
**Netrin-1 T7**			-	-		-	-	-	-	-	-	-
**- All**	0.524<br>0.021	F 470.87 vs M 425.22 p=0.014			-							
**- Real**	-	F= 473.47 vs M= 408.61 p=0.016			HT 408.61 vs no HT 473.47 0.009							
**- Sham**	-	-			-							
**Netrin-1 T14**	-	-	-	-		-	-	-	-	-	-	-
**- All**					HT 459.51 vs no HT 771.12 0.005							
**- Real**					-							
**- Sham**					-							
**BDNF T0**	-	-			-	-	-	-	-	-	-	-
**- All**			Obesity 17.87 vs no obesity 11.56 0.042									
**- Real**			-									
**- Sham**			Obesity 15.64 vs no obesity 11.0 0.040	Smoking 12.75 vs no smoking 10.28 p=0.086								
**BDNF T7**	-	-		-	-		-	-	-	-	-	-
**- All**			Obesity 16.41 vs no obesity 11.09 0.002			T7= 0.577, p=0.010 T14= 0.591, p=0.008						
**- Real**			-			-						
**- Sham**			-			-						

**BDNF T14**<br>**- All**<br>**- Real**<br>**- Sham**	-	-	-	-	-	-	-	-	-	-	-	-
**mir25 T0**<br>**- All**<br>**- Real**<br>**- Sham**	-	-	Obesity 0.22 vs no obesity 0.06 0.026<br>-<br>-	-	-	T7= 0.512, p=0.025 T14= 0.493, p=0.032<br>-<br>-	-	-	-	-	-	-
**mir25 T7**<br>**- All**<br>**- Real**<br>**- Sham**	0.457 0.049<br>-<br>-	-	Obesity 0.27 vs no obesity 0.05 0.033<br>-<br>-	-	-	T7= 0.598, p=0.007 T14= 0.577, p=0.010<br>-<br>-		IVT 0.18 vs no IVT 0.05 0.039	-	-	-	-
**mir25 T14**<br>**- All**<br>**- Real**<br>**- Sham**	-<br>0.681 P=0.030<br>-	-	-	0.640 0.004<br>-<br>-	0.513 0.030<br>-<br>-	T7= 0.595, p=0.009 T14= 0.583, p=0.011<br>T7= 0.685, 0.029 T14= 0.686, p=0.029<br>-	-	-	-	-	-	-
**mir93 T0**<br>**- All**<br>**- Real**<br>**- Sham**	0.562, p=0.012<br>-<br>-	-	-	-<br>-<br>Smoking 0.001 vs no smoking 0.03 p=0.027	-	T14= 9.534, p=0.019<br>-<br>-	-	-	-	-	-	-
**mir93 T7**<br>**- All**<br>**- Real**<br>**- Sham**	0.543 P=0.016<br>0.644 P=0.044<br>-	-<br>-<br>F 0.002 vs M 0.03 P=0.014	-	-	-	T14= 0.520, p=0.020<br>-<br>-	-	-	-	-	-	-
**mir93 T14**<br>**- All**		-	-	-	-	-	-	-	-	-	-	-

**- Real**<br>**- Sham**	0.510, p=0.030<br>-<br>-										-<br>Bridging 0.06 vs no bridging 0.003 p=0.046	
**mir106b T0**<br>**- All**<br>**- Real**<br>**- Sham**	-	-	-	-	-	T14= 0.528, p=0.024<br>-<br>-	-	-	-	-	-	-
**mir106b T7**<br>**- All**<br>**- Real**<br>**- Sham**	0.482 p=0.037<br>-<br>-	-	-	-<br>-<br>Smoking 0.002 vs no smoking 0.04 p=0.014	-	-	-	-	-	-	-	-
**mir106b T14**<br>**- All**<br>**- Real**<br>**- Sham**	-	-	-	-	-	-	-	-	-	-	-	-
**Semaphorin3A T0**<br>**- All**<br>**- Real**<br>**- Sham**	-	-	-	-	-	-	-	-	-	-	-<br>-<br>Bridging 40.02 vs no bridging 90.87 p=0.039	-
**Semaphorin3A T7**<br>**- All**<br>**- Real**<br>**- Sham**	 0.833 p=0.003 	-	-	-	-	T7= 0.506, p=0.027 T14= 0.541, p=0.017<br>T7= 0.838, p=0.002 T14= 0.898, p<0.001<br>-	-	-	TM 65.37 vs no TM 175.05 P=0.033<br>-<br>-	TM alone 65.37 vs no TM alone 175.05 p=0.033<br>-<br>TM alone 129.33 vs no TM alone 45.33 P=0.040	-	-
**Semaphorin3A T14**	-	-	-	-		-.		-	-	-	-	

**- All**					HT 67.15 vs no HT 184.47 p=0.027		-0.475 p=0.046					-
**- Real**					HT 55.25 vs no HT 184.47		-					-
**- Sham**					-							C=68.75 vs. S 193.74 vs CS 67.15 p=0.034

Numbers are: i) for continuous variables, Spearman's correlation coefficient (rho) and p values; ii) for dichotomous/categorical variables, median values and p values
C=cortical; CS=cortico-subcortical; F=female; HT= hemorrhagic transformation; IVT= intravenous thrombolysis; M=males; S=subcortical; TM=thrombectomy

Exploratory multivariate analysis

In the exploratory multivariate linear regression analysis for Ntn-1 and miR-25 levels at T14, rTMS resulted in being the only independent predictor in all three considered multivariate models model 1 adjusted for the main prespecified demographics and clinical variables and baseline (T0) Ntn-1 levels; model 2 and model 3 for other biomarkers which respectively, at T0 and T7, significantly correlated with T14 Ntn-1 levels, (B 179.55, Beta 0.676, 95% CI 67.47, 291.63, p=0.004 in all models) (Table S7.A). rTMS also resulted to be an independent predictor of the miR-25 levels at T14 in all three multivariate models (model 1: B 0.133, Beta 0.158, 95% CI 0.06, 0.21; p=0.002; models 2 and 3: B 0.127, Beta 0.271, 95% CI 0.09, 0.16, p<0.001) (Table S7.B).

Conversely, rTMS seems not to have an independent association with levels of miR-106b at T14 (Table S7.C).

Supplementary Table S7. Multivariate linear regression analyses for Netrin-1, miR25, and miR106b levels at T14

A. Netrin-1

	**B**	**Beta**	**95% CI**	**p**
**Model 1**				
rTMS (real vs. sham)	179.55	0.676	67.47, 291.63	0.004
**Model 2**				
rTMS (real vs. sham)	179.55	0.676	67.47, 291.63	0.004
**Model 3**				
rTMS (real vs. sham)	179.55	0.676	67.47, 291.63	0.004

Model 1: adjusted by rTMS (real vs sham), age, sex, baseline NIHSS, lesion volume, treatment with IVT and/or MT, baseline (T0) Netrin-1 levels

Model 2: adjusted by rTMS (real vs sham), age, sex, baseline NIHSS, lesion volume, treatment with IVT and/or MT, baseline (T0) Netrin-1 levels, BDNF T0, Semaphorin 3A T0

Model 3: adjusted by rTMS (real vs sham), age, sex, baseline NIHSS, lesion volume, treatment with IVT and/or MT, baseline (T0) Netrin-1 levels, BDNF T0, mir25 T7, Semaphorin 3A T0, Semaphorin 3A T7.

B. miR25

	**B**	**Beta**	**95% CI**	**p**
**Model 1**				
rTMS (real vs. sham)	0.133	0.158	0.06, 0.21	0.002
miR-25 T0	1.739	0.920	1.57, 1.91	<0.001
Sex (males)	-0.073	-0.087	-0.145, -0.002	0.046
**Model 2**				
rTMS (real vs. sham)	0.127	0.271	0.09, 0.16	<0.001
miR-25 T0	1.391	0.817	1.22, 1.56	<0.001
miR-106b T0	0.072	0.190	0.04, 0.11	0.001
BDNF T0	-0.006	-0.120	-0.011, -0.002	0.012
**Model 3**				
rTMS (real vs. sham)	0.127	0.271	0.09, 0.16	<0.001

miR-25 T0	1.391	0.817	1.22, 1.56	<0.001
miR-106b T0	0.072	0.190	0.04, 0.11	0.001
BDNF T0	-0.006	-0.120	-0.011, -0.002	0.012

Model 1: adjusted by rTMS (real vs sham), age, sex, baseline NIHSS, lesion volume, treatment with IVT and/or MT, baseline (T0) miR-25 levels
Model 2: adjusted by rTMS (real vs sham), age, sex, baseline NIHSS, lesion volume, treatment with IVT and/or MT baseline (T0) mir25 levels, BDNF T0, miR-106b T0, Semaphorin 3A T0
Model 3: adjusted by rTMS (real vs sham), age, sex, baseline NIHSS, lesion volume, treatment with IVT and/or MT baseline (T0) miR-25 levels, miR-25 T7, BDNF T0, mir-106b T0, Semaphorin 3A T0, Semaphorin 3A T7; results are the same also after inclusion of NIHSS T7 in the model 3.

C. miR-106b

	**B**	**Beta**	**95% CI**	**p**
**Model 1**				
miR-106b T0	1.650	0.756	0.793, 2.506	0.001
**Model 2**				
miR-106b T0	0.928	0.733	0.891, 0.964	<0.001
miR-93 T0	0.263	0.425	0.257, 0.269	<0.001
**Model 3**				
miR-106b T0	0.928	0.733	0.891, 0.964	<0.001
miR-93 T0	0.263	0.425	0.257, 0.269	<0.001

Model 1: adjusted by rTMS (real vs sham), age, sex, baseline NIHSS, lesion volume, treatment with IVT and/or MT, baseline (T0) miR-106b levels**.**
Model 2: adjusted by rTMS (real vs sham), age, sex, baseline NIHSS, lesion volume, treatment with IVT and/or MT, baseline (T0) miR-106b levels, miR-93 T0, Semaphorin 3A T0
Model 3: adjusted by rTMS (real vs sham), age, sex, baseline NIHSS, lesion volume, treatment with IVT and/or MT, baseline (T0) miR-106b levels, miR-106b T7, miR-93 T0, miR-93 T7, Semaphorin 3A T0.

Supplementary Figure S1. Correlations between netrin-1 and mir25 at T0 (A) and T7 (B) in All patients (real+sham) and at T14 in real and sham (C) as separate subgroups

A. All at T0

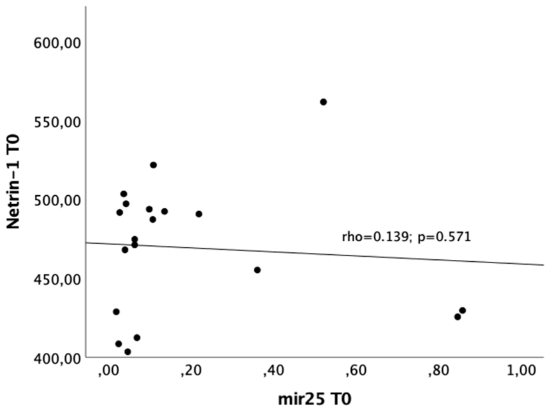

B. All at T7

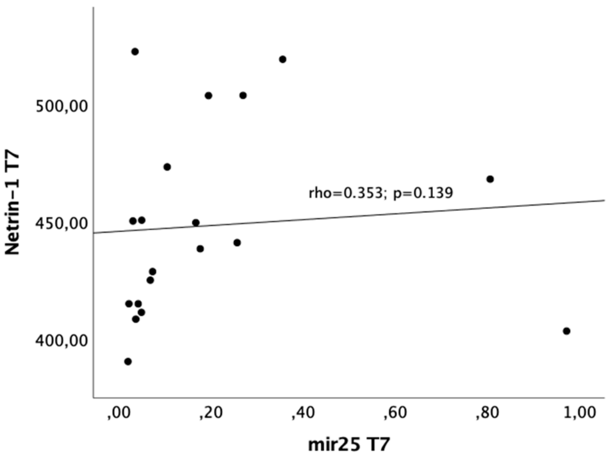

C. Real and sham as separate subgroups at T14

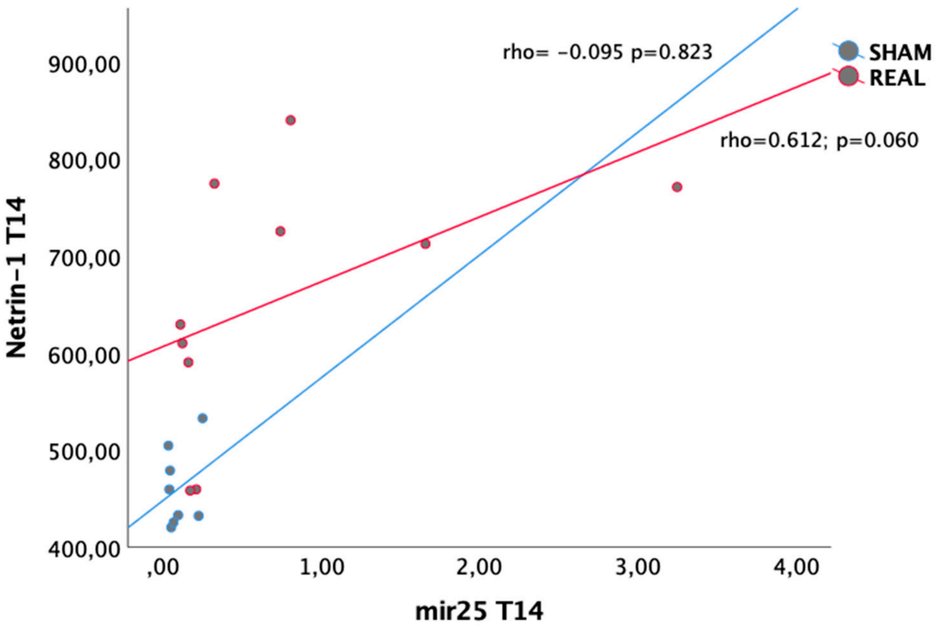

Supplementary Figure S2. Correlations between mir25 and BDNF at T0 (A) and T7 (B) in All patients (real+sham) and at T14 in real and sham (C) as separate subgroups

A. All at T0

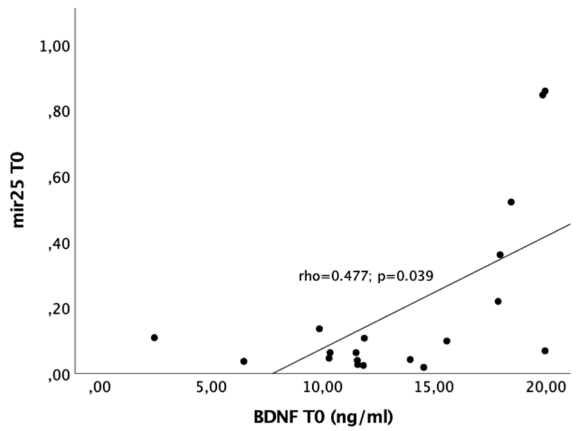

B. All at T7

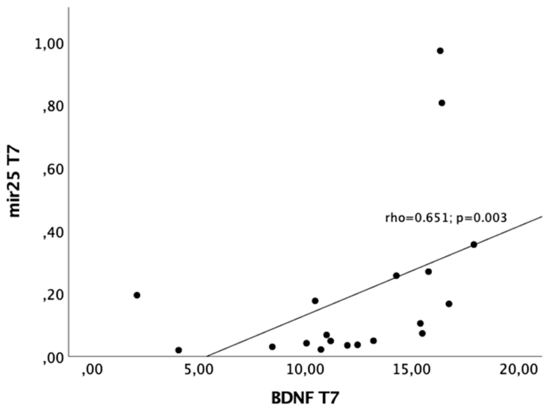

C. Real and sham as separate subgroups at T14

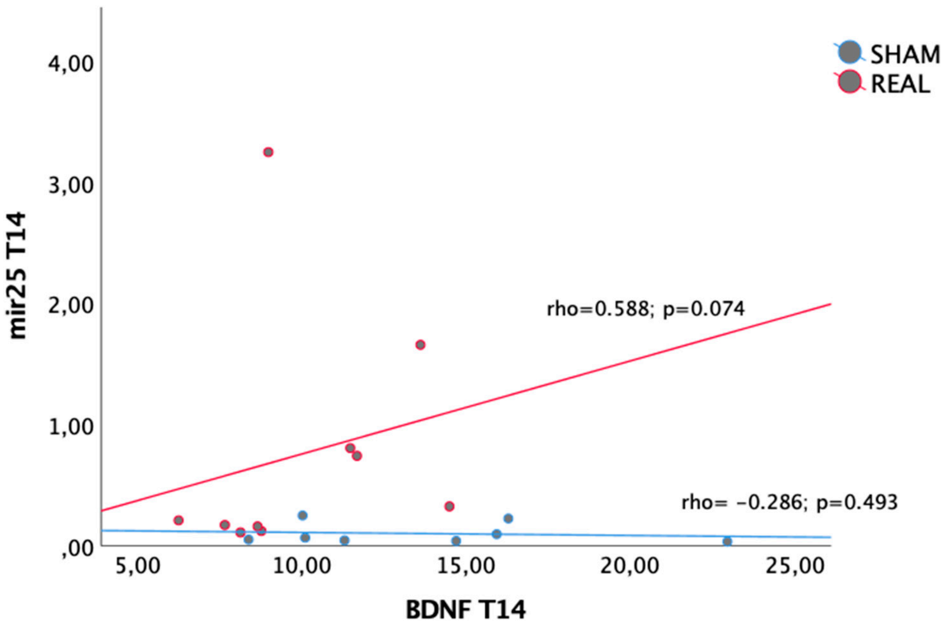

Supplementary Figure S3. Correlations among mir25, mir93, and mir106b at T0 (A) and T7 (B) in All patients (real+sham) and at T14 in real (C) and sham (D) subgroups

A. All at T0

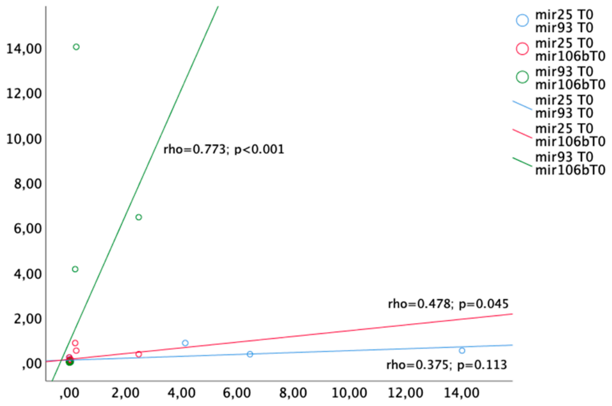

B. All at T7

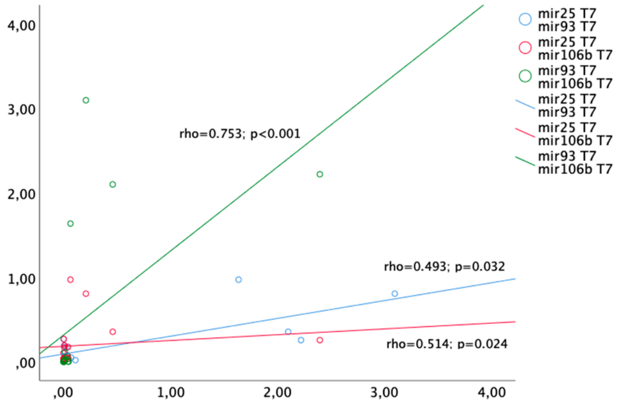

C. Real at T14

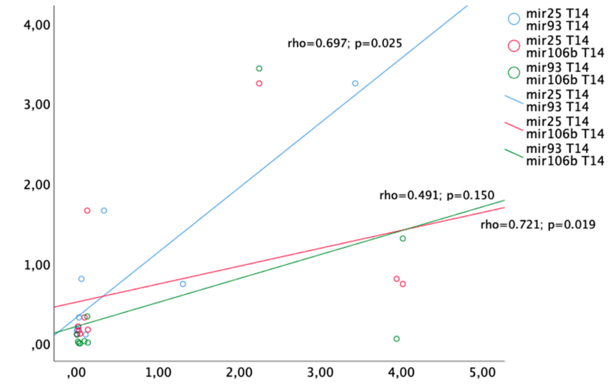

D. Sham at T14

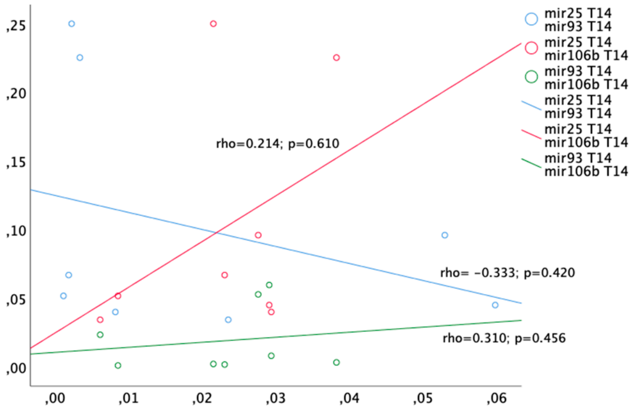

Supplementary Figure S4. Correlations between netrin-1 and semaphorin3A as axonogenesis molecular biomarkers at T0 (A) and T7 (B) in All patients (real+sham) and at T14 in real (C) and sham (D) subgroups

A. All at T0

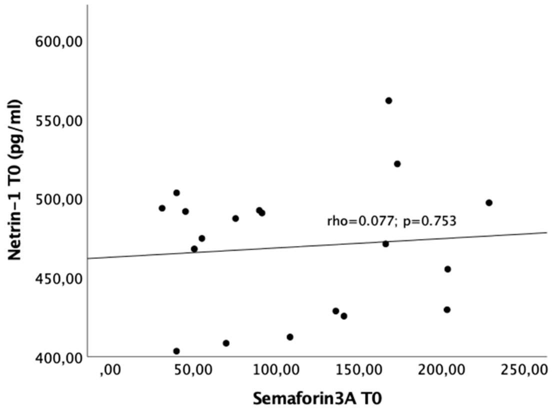

B. All at T7

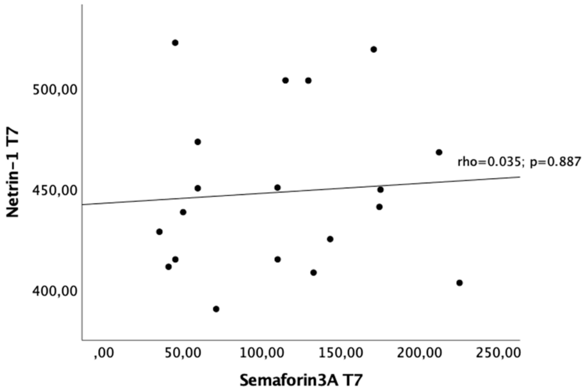

C. Real and sham as separate subgroups at T14

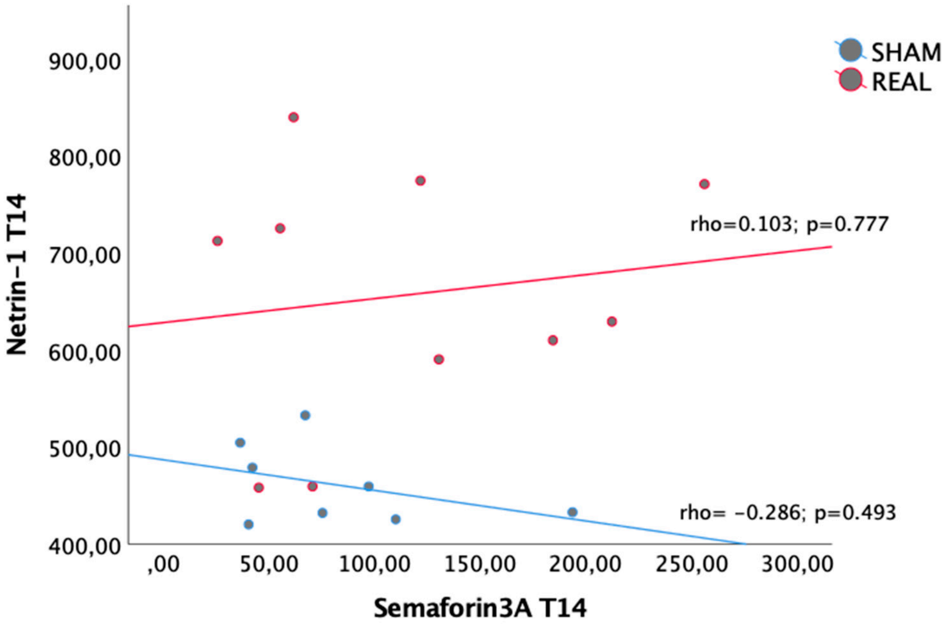

Supplementary Figure S5. Statistically significant correlations between age (A) and NIHSS at T7 (B) and molecular neurogenesis biomarkers (all with p<0.05)

A.

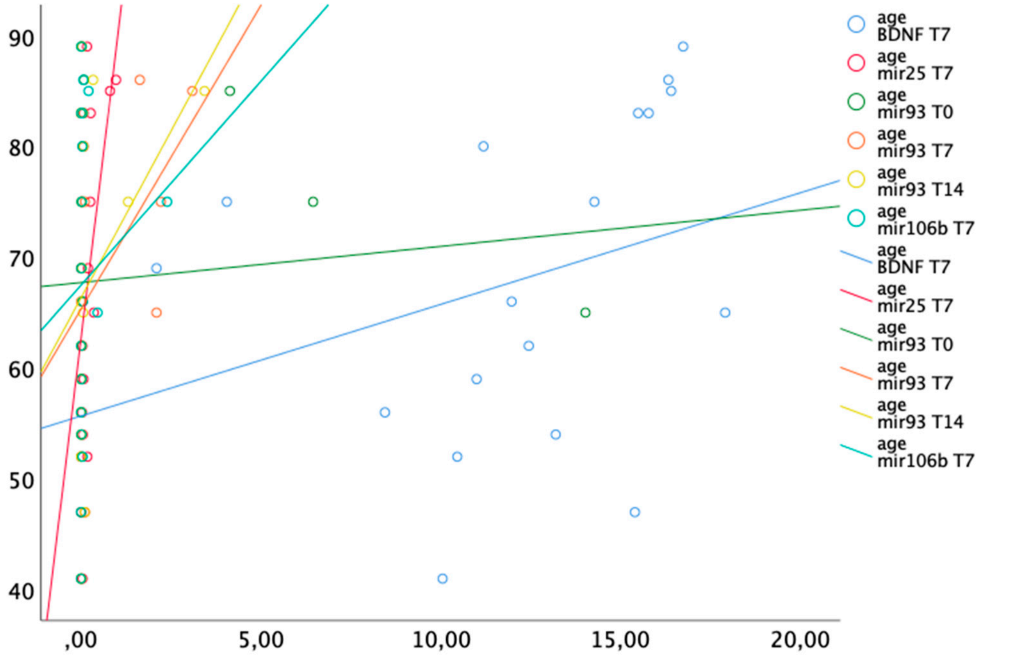

B.

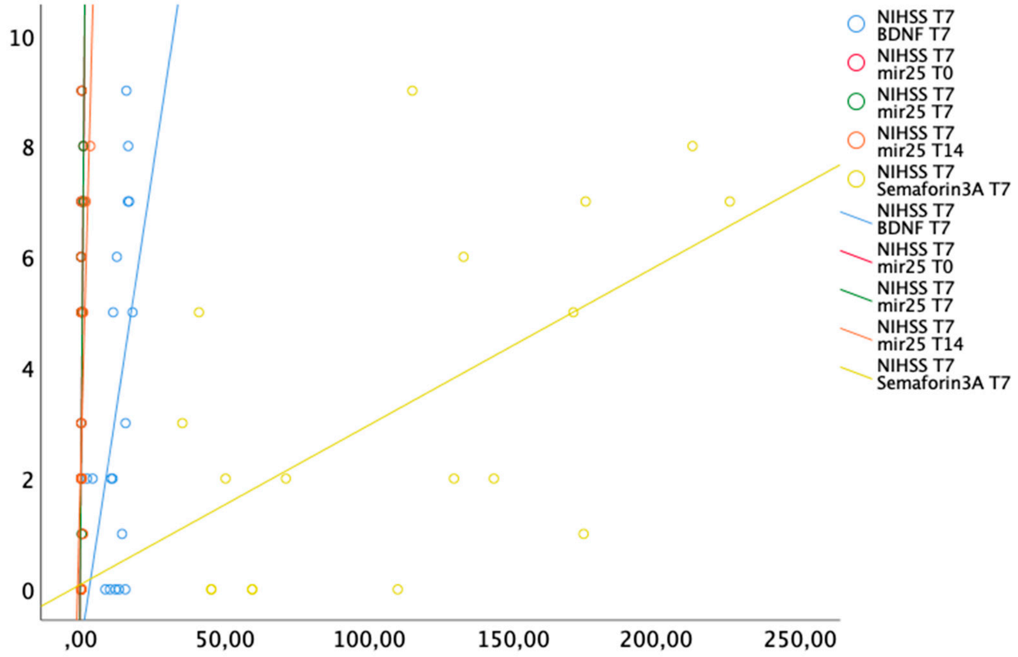

Supplement: Supplementary file 1 [file biomedicines-12-00670-s001.zip › biomedicines-2912927-supplementary.pdf]
